# Supplementary material for: Synthetic torpor in the rat protects the heart from ischaemia-reperfusion injury
Source: eLife. 2026 Nov 12;15:RP110559. doi: 10.7554/eLife.110559 (PMC13759185; doi:10.7554/eLife.110559)
Supplement: Peer review history

# Editor report: eLife Assessment

**DOI:** 10.7554/eLife.110559.3.sa0

**Authors:** Moses V Chao

**Affiliations:** NYU Langone Health United States

**Keywords:** Important, Solid

This **important** submission from Ambler and colleagues brings new insights into how torpor conditions may confer resilience in cases of cardioprotection. The study is backed by **solid** evidence and represents a unique interoceptive mechanism of interest.

# Referee report: Reviewer #1 (Public review)

**DOI:** 10.7554/eLife.110559.3.sa1

[Editors' note: this version has been assessed by the Reviewing Editor without further input from the original reviewers. After previous evaluation by three competent reviewers, the authors have provided an extensive response to their criticisms and recommendations. I believe that authors have done an adequate job in addressing the initial reviews and recommend that the paper be processed for the Version of Record without further work at this time.]

Summary:

Torpor can be induced by chemogenetic activation of the medial preoptic area. This activation leads to protection from myocardial infarction in an isolated heart preparation despite normalization of the ambient temperature, thus, in principle, uncoupling hypothermia from torpor-induced neuroprotection. Putative pathways of protection are suggested by proteomic studies.

Strengths:

(1) Elegant strategy for inducing torpor in rats.

(2) Appropriate controls for verifying the neuron transducer.

(3) Cardiac protection is significant and appears independent of hypothermia.

(4) Interesting omic strategy to begin to find established and novel pathways mediating organ autonomous torpor-induced protection.

# Referee report: Reviewer #2 (Public review)

**DOI:** 10.7554/eLife.110559.3.sa2

Summary:

Elley and colleagues induced a synthetic torpor-like state in rats (a non-hibernating species) by chemogenetically activating neurons in the medial preoptic area of the hypothalamus. They show that this state substantially reduced cardiac infarct size in an ex vivo ischemia-reperfusion model. They further report that protection persisted when ambient temperature was raised to prevent hypothermia and used exploratory phosphoproteomics to identify candidate cardioprotective signaling pathways.

Strengths:

This is the first demonstration that a torpor-like state is cardioprotective in a species that does not naturally enter torpor, which meaningfully advances the potential clinical utility of synthetic torpor. The experimental design is logical, and the controls are generally appropriate. The characterisation of the responsible neuronal population using ISH against QPLOT markers adds mechanistic depth and supports the cross-species conservation argument. The phosphoproteomic analysis, though exploratory, generates plausible and biologically coherent hypotheses grounded in the hibernation literature.

# Referee report: Reviewer #3 (Public review)

**DOI:** 10.7554/eLife.110559.3.sa3

Summary:

The manuscript by Elley and colleagues describes experiments on the effects of synthetic torpor on ex vivo heart ischemia. The key aspect of the study was the use of viral-vector mediated manipulation of the hypothalamic medial preoptic area (MPA) in rats. They used AAV-CaMKIIa-hM3D(Gq). The authors report that chemogenetic activation of the MPA prior to an ex vivo heart ischemia-reperfusion insult induces cardio protection against infarct size that is independent of prior in vivo hypothermia. Phosphoproteomic analysis of cardiac tissue suggested changes in cell survival and death pathways.

Strengths:

This study has important strengths. The idea is novel. The experimental design is appropriately rationalized and fascinating. The manuscript is written and presented concisely.

# Referee report: Reviewer #4 (Public review)

**DOI:** 10.7554/eLife.110559.3.sa4

**From the Reviewing Editor:**

The core finding and implication of this article is that reduced body temperature and hibernation conditions may have value in understanding basic physiology and during medical conditions. The subject of this submission should be considered as a new interoception topic, which is of current high interest that promises to link CNS regulatory mechanisms to the cardiovascular system and vice versa. But there is also substantial relevance to sleep, the immune system, cancer and protective mechanisms to other organ systems after injury and disease.

This submission demonstrates that a torpor-like state can be related to a cardioprotective effect in a model of ischemia-reperfusion injury. Torpor is characterized by hyperthermia and reduced heart rate and oxygen consumption. As such, it has complicated and unpredictable physiological impacts upon a variety of organ systems. The study contends that the effect of torpor is mediated by the medial preoptic region of the hypothalamus. Protein analysis in a rat model suggests the effects of a torpor-like state may involve detectable effects upon intrinsic cell death and stress related pathways. A further understanding the mechanisms of torpor research will likely reveal new protein and genetic targets that are conserved targets. The authors propose a hypothesis that torpor conditions may reflect an evolutionary mechanism that will have implications for considering application to clinical trials and therapeutic protocols.

This is a provocative study that raises many interesting questions and suggestions for further study. For the most part, the authors acknowledge that there are certainly many future experiments that should be carried out on the basis of the results from the current study. Notwithstanding the need for more experimentation to extend our understanding of torpor, the authors respond to the reviews with additional supplementary data and the recognition of important caveats for alternative brain and physiological mechanisms in play.

# Author comment: Author response

**DOI:** 10.7554/eLife.110559.3.sa5

**Authors:** Megan Elley, Ludovico Taddei, Muzammil Ali Khan, Una R Wilcox, Timna Hitrec, Martin Lewis, Anthony E Pickering, Michael Thomas Ambler

**Affiliations:** University of Bristol Bristol United Kingdom; University of Bristol Bristol United Kingdom; University of Bristol Bristol United Kingdom; Queen Mary University of London London United Kingdom; University of Bologna Bologna Italy; University of Bristol Bristol United Kingdom; University of Bristol Bristol United Kingdom; University of Bristol Bristol United Kingdom

The following is the authors’ response to the original reviews.

**Public Reviews:**

**Reviewer #1 (Public review):**

Torpor can be induced by chemogenetic activation of the medial preoptic area. This activation leads to protection from myocardial infarction in an isolated heart preparation despite normalization of the ambient temperature, thus, in principle, uncoupling hypothermia from torpor-induced neuroprotection. Putative pathways of protection are suggested by proteomic studies.

Strengths:

(1) Elegant strategy for inducing torpor in rats.

(2) Appropriate controls for verifying the neuron transducer.

(3) Cardiac protection is significant and appears independent of hypothermia.

(4) Interesting omic strategy to begin to find established and novel pathways mediating organ autonomous torpor-induced protection.

We thank the reviewer for their positive feedback and review of our study.

Weaknesses:

(1) The study would benefit from using inhibitory chemogenetics of the same neurons to demonstrate that this might make cardiac response to ischaemia worse.

This is an interesting experiment proposal and is something we will consider for the future. It is not immediately clear to us whether the hypothesis would be that inhibiting these neurons would make cardiac ischaemia worse, or that it would be similar to the control groups as we would not be inducing synthetic torpor.

(2) Infecting an area of the brain not known to be involved in torpor would be a useful control.

We chose to infect neurons in the same brain region without the presence of the HM3DGq DREADD construct as our control. This is a well-established control for this type of experiment.

(3) In vivo cardio protection seems essential as the validation of the strategy requires support that is in the intact animal.

We agree that an *in-vivo* model is important to establish whether the same cardioprotection occurs in the intact animal and whether it is observed when synthetic torpor is induced following an ischaemic injury. Our data suggests that synthetic torpor pre-conditions the cardiac tissue, prior to isolation of the heart, as the protection remains post removal of the heart and without any ongoing *in-vivo* systemic signal/drive. This provides some evidence that this may be used as a pre-conditioning model for organ protection.

(4) The assumption that the positive effects of torpor are mediated via a phosphoproteomic change rather than a translational or transcriptional control mechanism is not established.

Cardiac tissue for the proteomics experiment was collected following 90 minutes of synthetic torpor. This time point was selected for several reasons, including: *in-vivo* body temperature and heart rate reach their nadir at this time point, and we collected the hearts for the Langendorff ischaemia-reperfusion injury data set at 90 minutes and wanted to keep this consistent.

While we tested for both total protein and phosphoprotein changes, the only significant differences occurred in the phosphoprotein levels. At 90 minutes, the predominant mechanisms for cellular responses are phosphorylation changes – somewhat too early for changes in transcription or translation. In future, it would be of interest to look at later time points to determine whether changes have occurred at the transcription/translation level.

(5) A 40 percent reduction in infarct size may work for genetically identical rats with no co-morbidities, but is unlikely to be significant enough to weather the variability that emerges in humans because of these differences and more. The question is not what the mechanism is, but how do we make it more robust? Overall, this is at best a preliminary data set that requires more experiments to deliver on its immense promise.

We agree with the reviewer that these are preliminary findings, as this is the first demonstration of a torpor-like state in a species that does not naturally enter torpor is protective. However, understanding the mechanisms by which the cardioprotection occurs is going to be important if we want to replicate this state in humans and to understand whether this is specific to cardiac tissue or if it extends to multiple different organs. Furthermore, by understanding the mechanisms, this may reveal avenues that would allow us to enhance these protective effects and make the method more robust.

**Reviewer #2 (Public review):**

Summary:

Elley and colleagues induced a synthetic torpor-like state in rats (a non-hibernating species) by chemogenetically activating neurons in the medial preoptic area of the hypothalamus. They show that this state substantially reduced cardiac infarct size in an ex vivo ischaemia-reperfusion model. They further report that protection persisted when ambient temperature was raised to prevent hypothermia, and used exploratory phosphoproteomics to identify candidate cardioprotective signaling pathways.

Strengths:

This is the first demonstration that a torpor-like state is cardioprotective in a species that does not naturally enter torpor, which meaningfully advances the potential clinical utility of synthetic torpor. The experimental design is logical, and the controls are generally appropriate. The characterisation of the responsible neuronal population using ISH against QPLOT markers adds mechanistic depth and supports the cross-species conservation argument. The phosphoproteomic analysis, though exploratory, generates plausible and biologically coherent hypotheses grounded in the hibernation literature.

We thank the reviewer for their positive assessment of our work.

Weaknesses:

The primary weakness is that the central conclusion - that hypothermia is not necessary for cardioprotection - exceeds the evidence. The thermoneutral groups were not demonstrably normothermic (36.4 vs 37.05{degree sign}C, p=0.44 with n=6), core temperature telemetry was absent in the majority of control animals contributing to the infarct endpoint, and the decisive test, i.e., a correlation between individual nadir temperature and infarct size, was never performed. Additional weaknesses include the absence of sex-stratified analysis despite known estrogenic contributions to torpor

The core body temperature in the thermoneutral synthetic torpor animals was ~ 0.6°C cooler than the control animals housed at room temperature, which was not significantly different. A 0.6°C body temperature reduction is akin that the changes observed around a sleep-wake cycle and we consider it to be biologically implausible that this degree of temperature reduction in the ‘thermoneutral synthetic torpor’ group is sufficient to account for the observed cardioprotection. We have since added a graph and correlation analysis of individual surface temperature against infarct size in the synthetic torpor animals (see supplementary figure 5). This demonstrates no correlation between nadir temperature during synthetic torpor and infarct size, supporting the hypothesis that the protective effect is not driven by cooling.

**Reviewer #3 (Public review):**

Summary:

The manuscript by Elley and colleagues describes experiments on the effects of synthetic torpor on ex vivo heart ischaemia. The key aspect of the study was the use of viral-vector mediated manipulation of the hypothalamic medial preoptic area (MPA) in rats. They used AAV-CaMKIIa-hM3D(Gq). The authors report that chemogenetic activation of the MPA prior to an ex vivo heart ischaemia-reperfusion insult induces cardio protection against infarct size that is independent of prior in vivo hypothermia. Phosphoproteomic analysis of cardiac tissue suggested changes in cell survival and death pathways.

Strengths:

This study has important strengths. The idea is novel. The experimental design is appropriately rationalized and fascinating. The manuscript is written and presented concisely.

We thank the reviewer for their positive evaluation of our paper.

Weaknesses:

The study has important weaknesses in the experimental design and validation of the model.

(1) The study is based on the use of a DREADD-designed viral vector (AAV-CaMKIIa-hM3D(Gq) -mCherry) that is activated by 2 mg/kg IP injection of CNO. The rationale is to putatively activate the MPA. The authors show no evidence for chemogenetic activation of neurons in the MPA. This could be done using a variety of different approaches, even phosphoproteomics.

Chemogenetics is a very well-established technique and we demonstrate histological data showing expression of the DREADD in the MPA region and large physiological changes in body temperature, heart rate and oxygen consumption that we feel is sufficient to demonstrate that these neurons are activated. The field of chemogenetics is sufficiently established that it is not common practice to demonstrate neuronal activation in the context of large induced physiological responses.

(2) The stereotaxic injections are difficult to precisely and locally place, particularly bilaterally. Figure 2F is only a schematic. It would be better to show actual low magnification brain sections (bregma +0.12 to -0.48) from a representative rat to show the placement of the AAV.

Figure 2F is a schematic produced from imaging data, showing the regions within the MPA that expressed the AAV across all animals. We mapped the areas of viral transfection in each animal and then mapped this onto the brain atlas demonstrating which regions were consistently transduced across all animals. We have added a supplementary figure (supplementary figure 2) showing overlaid sections from 6 animals that underwent detailed vector mapping, which shows the full extent of vector transduction in these animals.

(3) The control rats were injected with AAV-CaMKIIa-EGFP. Why was EGFP used instead of mCherry for the control?

We prioritised using the same serotype and promoter for our experiments and would not anticipate the colour of fluorophore to have an effect on experimental results.

(4) Ideally, a mutant non-activatable variant of AAV-CaMKIIa-hM3D(Gq) should have been used for a better control.

We thank the reviewer for this suggestion, however, to the best of our knowledge, a viral vector supplier Addgene, does not stock this variant of virus, nor have we seen one reported in the literature.

(5) The authors should comment on whether there is any neurotoxicity in the MPA associated with the forced AAV expression of hM3D-Gq.

This is not something we have assessed, however, administration of AAV into the CNS is not associated with a robust inflammatory response or neurotoxicity [1]. We used a viral titre of 1.7x10^13^ viral genome copies per ml and injected 200 nL at each POA site, which is within reported guidelines by other users of the Addgene construct.

(1) Mastakov MY, Baer K, Symes CW, Leichtlein CB, Kotin RM, During MJ. Immunological aspects of recombinant adeno-associated virus delivery to the mammalian brain. J Virol. 2002;76(16):8446-8454. doi:10.1128/jvi.76.16.8446-8454.2002

(6) Is there any inflammatory pathology seen in the MPA with AAV transduction?

This is not something that we assessed.

(7) There are no experiments to show that the systemic torpor is specifically associated with the MPA region. Experiments should be done with injections of AAV-CaMKIIa-hM3D(Gq)-mCherry placed in other brain regions, for example, the nearby nucleus accumbens.

The medial preoptic area is widely believed to be key for triggering torpor. Data from our lab and other groups has demonstrated that neurons within the preoptic area are sufficient to trigger torpor entry in mice. However, we concede that it is possible that other brain regions form part of the same circuit and that activation of the circuit at this / these points could also trigger a synthetic torpor bout. Should this be the case, our key message would remain the same: animals that do not naturally enter torpor can be induced into a similar state through targeted CNS activation, and this state is cardioprotective.

(8) The mapping of the distribution of neurons responsible for synthetic torpor is not mechanistic enough and is not directly to the point. While excitatory and inhibitory markers are examined, a more interesting and deeper approach would have been to use glutamate receptor antagonists to manipulate the torpor response.

We thank the reviewer for this interesting suggestion. This type of experiment is not commonly performed and would be extremely technically challenging. A more common approach would be to genetically access glutamatergic neurons in the region and silence them with chemo or optogenetics.

(9) The ischaemia and reperfusion aspects of the Langendorff method need to be clarified. The isolated hearts are already ischaemic after their removal from the rat. The reperfusion aspect is caused by reflow of blood to generate oxidative stress, but in the ex vivo model, is there really reperfusion injury?

The Langendorff model is a well-established *ex-vivo* technique where the excised heart is perfused retrogradely through the aorta. This allows oxygenated buffer that also contains metabolic fuel to be pumped through the aorta, forcing the aortic valve closed and diverts the buffer directly into the coronary arteries to sustain the organ outside of the body. By temporarily stopping buffer perfusion then reinstating it as we did here, we can mimic a full ischaemia-reperfusion injury. It has been commonly used since the late 19th century to study heart physiology and pathophysiology including ischaemia-reperfusion injury.

We agree with the reviewer that the hearts may have experienced a degree of ischaemia when being removed, unfortunately that is one of the confounds associated with *ex-vivo* preparations such as the Langendorff model. However, it should be noted that both the control and synthetic torpor hearts were exposed to the same conditions following removal from the animal. To minimise ischaemic damage, all hearts were placed in ice-cold buffer immediately following excision. Preparations were excluded from our data if (a) cannulation took longer than 3 minutes, (b) more than 2 cannulation attempts were made and (c) if the heart rate was slower than 200 bpm during the baseline 30-minute recording. This criteria is in accordance with other laboratories and is frequently reported in the literature.

(10) The authors show that whole animal oxygen consumption is reduced in the torpor state. The measurement is crude and most likely reflects the inactivity of the animal's skeletal muscle in the torpor state. A more relevant and direct experiment would be to do oxygen consumption (or Seahorse) assays on extracts of the isolated hearts.

We did not simultaneously measure oxygen consumption alongside animal activity so we cannot determine whether muscle inactivity is a contributing factor in this data. However, previous work on mice has shown that VO_2_ to be decreased by 15% during the light phase (mouse inactivity/sleep period) compared to the dark phase (their active phase) [1]. Whereas during fasting-induced torpor, a ~45% decrease in VO_2_ has been reported [2], which is consistent with what we observed during synthetic torpor in rats (~39% decrease in VO_2_).

Furthermore, we simultaneously recorded activity of our rats while recording core body temperature and heart rate before and during synthetic torpor. We have since updated the manuscript to include this data as a supplementary figure (supplementary figure 6), showing that there is no significant difference between the activity of animals in synthetic torpor compared to controls (a curious contrast with natural torpor).

We also agree with the reviewer regarding the seahorse experimental suggestion and plan to use the seahorse assay to measure oxygen consumption in isolated cardiomyocytes and cardiac tissue punches in the future.

(1) Nie Y, Gavin TP, Kuang S. Measurement of Resting Energy Metabolism in Mice Using Oxymax Open Circuit Indirect Calorimeter. Bio Protoc. 2015;5(18):e1602. doi:10.21769/bioprotoc.1602

(2) Hrvatin S, Sun S, Wilcox OF, et al. Neurons that regulate mouse torpor. Nature. 2020;583(7814):115-121. doi:10.1038/s41586-020-2387-5

(11) The authors report that the synthetic torpor induces bradycardia. There is no follow-up on this important observation. The MPA-heart connection is not analyzed. (A) Is the link through cardiovascular centers in the brainstem? (B) Is the torpor-induced bradycardia mediated through increased parasympathetic or decreased sympathetic autonomic tone? Pharmacological experiments could also be done.

We thank the reviewer for these suggestions and agree that these will be important follow-up experiments to understand how the cardioprotection is mediated. We currently have a grant under review that will perform these important experiments.

**Recommendations for the authors:**

**Reviewer #1 (Recommendations for the authors):**

Suggestions for improved or additional experiments or analysis:

(1) Chemogenetic inhibition of the MPO.

This is an interesting experiment proposal, we chose to focus on the activation of these neuronal populations to establish whether activation would be sufficient to drive synthetic torpor in a species that does not naturally enter torpor. Inhibiting these neurons would be an interesting avenue to explore in the future.

(2) In vivo validation of torpor-induced protection.

We plan to use an *in-vivo* cardiac ischaemia-reperfusion injury model in future experiments and this forms a work package in a grant we currently have under review.

(3) A search for the dose and timing of torpor that provides the most robust protection.

Using the *in-vivo* cardiac ischaemia-reperfusion injury model described above, in future we aim to determine whether synthetic torpor is protective following the ischaemic episode, which would better model a clinical scenario such as when a patient presents with a heart attack.

(4) Use of inhibitors of transcription and translation as a strategy to understand what are the nodes of regulation that mediate neuroprotection.

An interesting suggestion. We might consider this in future studies but feel it is beyond the scope of this manuscript.

(5) Testing in males, females, and rats of different genetic backgrounds.

This data set includes both male and female rats, however we did not power our experiments to determine sex differences. In future, we will plan to power our studies to allow determination of sex differences, and to use rats from different genetic backgrounds.

(6) Use of chemical or molecular tools to assess the relevance of phosphoproteomic data.

Our future experimental plans, which are currently under review in a grant application include the use of inhibitors and activators of the kinases we identified in our phosphoproteomic data, to determine whether these can modulate the infarct size in the ischaemia-reperfusion injury model and modulate the cardioprotective effect from synthetic torpor.

**Reviewer #2 (Recommendations for the authors):**

(1) Clarifying core body temperature measurement:

The authors report post-CNO core body temperature (mean, 90-100 minute window) for four groups: room temperature MPAGq (31.64 {plus minus} 1.02{degree sign}C, n=10), room temperature MPAEGFP (37.05 {plus minus} 0.43{degree sign}C, n=6), thermoneutral MPAGq (36.4 {plus minus} 0.73{degree sign}C, n=6), and thermoneutral MPAEGFP (37.05 {plus minus} 0.43{degree sign}C, n=6). Please clarify the provenance of the MPAEGFP temperature data, as the room temperature and thermoneutral MPAEGFP groups report identical means to two decimal places (37.05{degree sign}C) with identical standard deviations ({plus minus}0.43{degree sign}C). It should also be noted in the limitations that continuous core temperature telemetry was available in only a subset of animals - specifically, 10 of 12 room temperature MPAGq animals and 6 of 16 room temperature MPAEGFP animals that contributed to the primary Langendorff infarct endpoint. It is correct that this means that for the majority of room temperature MPAEGFP control animals (10 of 16), no core temperature measurement of any kind was paired with their infarct data? If so, this should be stated as a limitation. Additionally, pre-CNO baseline temperatures are not reported numerically for any group, and individual nadir temperatures across the 90-minute induction period are not provided, meaning the full thermal exposure of individual animals cannot be characterized from the data as presented.

We thank the reviewer for these suggestions, and we have since corrected the in-text error for the MPA^EGFP^ thermoneutral and room temperature data. While we did not have the *in-vivo* core body temperature in all of our animals, we did record surface body temperature if core body temperature was absent and we have since plotted this surface body temperature data against the infarct size and performed a correlational analysis. We found no significant correlation between the two. We have since also updated our supplementary figure 1 to include the pre-CNO baseline temperature and heart rates for all animals in the synthetic torpor and control groups and those exposed to room temperature or thermoneutral environments.

(2) Conclusions exceed evidence:

While the demonstration that chemogenetic MPA activation under thermoneutral conditions is associated with reduced infarct size is an interesting and valuable finding, the conclusion, as stated, that "the hypothermic component of torpor is not necessary for the protective effects of synthetic torpor" (lines 303-304), goes beyond what the evidence can support. The cardioprotective effect observed in the thermoneutral condition may indeed reflect the contribution of other (non-temperature) factors - but the involvement of a temperature effect cannot be ruled out on the basis of the data presented. The thermoneutral MPAGq group, although not significantly different from the thermoneutral MPAEGFP control in core temperature (36.4 {plus minus} 0.73{degree sign}C vs. 37.05 {plus minus} 0.43{degree sign}C, p=0.44), had a mean core temperature that was in fact 0.65{degree sign}C lower than controls, and with n=6 in each group the non-significance of this difference reflects limited statistical power to detect equivalence as much as true normothermia. Given the limitations in telemetric coverage described above, a non-significant difference between group means is not sufficient to conclude that hypothermia is categorically uninvolved in mediating the cardioprotective effect. The appropriate test would be to demonstrate an absence of correlation between individual core body temperature nadir and individual infarct size across all animals in both the room temperature and thermoneutral conditions - a continuous analysis that the current telemetry coverage may not fully support, but that would directly address the question. We would therefore ask the authors either to provide such a correlation analysis if the paired data permit it, or to temper their conclusion accordingly - stating that factors other than hypothermia are sufficient for cardioprotection, rather than that hypothermia is not involved, which is the stronger and less well-supported claim.

We agree with these suggestions and have adjusted our conclusions within the text. We have also performed a correlational analysis between the surface temperature of the animals and the infarct area, which we have included in the supplementary figures. We found no significant correlation between the two, which further supports our conclusion that other factors are likely to contribute to the cardioprotection observed, rather than the hypothermia. We feel that it is biologically implausible to suppose that the 0.6°C drop in body temperature observed in the thermoneutral synthetic torpor group is sufficient to recapitulate the cardioprotection observed.

(3) Use of the word torpor: The statement:

"this cardioprotection induced by synthetic torpor persisted in the absence of hypothermia"

Presupposes that the thermoneutral condition is still synthetic torpor, just without one feature. But the authors have no basis for that framing - they have not demonstrated that the thermoneutral condition preserves the metabolic suppression component, and they have in fact abolished the temperature component that is part of the very definition they invoke. A more accurate statement would be:

"Chemogenetic activation of MPA neurons under thermoneutral conditions, which prevents hypothermia and whose effect on oxygen consumption was not assessed, still produced a reduction in infarct size."

We thank the reviewer for this suggestion, and we have amended the text in our manuscript to reflect this.

(4) Lack of data addressing possible sex differences. Given the known role of estrogen in contributing to torpor, a more thorough analysis of sex differences beyond underpowered stratification is needed. In the absence of this analysis, this should be noted as a limitation.

The preoptic area of the hypothalamus is enriched with estrogen receptors and previous work by our group has demonstrated that the estrous cycle modulates fasting-induced torpor. Our current work did not aim to investigate sex differences and is not powered to do this. As a result, we have now added this limitation to our discussion.

(5) QPLOT marker characterization is incomplete. The QPLOT classification requires co-expression of markers including Qrfp and Tacr3, but the authors only tested three of the five (Ptger3, Lepr, Opn5). Ptger3 was expressed in 100% of transduced cells, but the question is whether Ptger3 expression alone is sufficient evidence of QPLOT identity without testing Qrfp and Tacr3. The conclusion that these neurons are "analogous" to mouse QPLOT neurons is reasonable but somewhat circular, given the marker selection. The assertion that 29% expressed "all three QPLOT markers" should be contextualized as three of five.

We have updated our manuscript to state that our assessment is for three of the five QPLOT neuron markers.

**Reviewer #3 (Recommendations for the authors):**

Other comments:

(12) The figure legends should state the group sizes (animal numbers) for the graphical data.

We have added this information to the figure legends.

(13) The control and experimental groups do not appear to be balanced. Were any animals removed from the treatment groups?

Some MPA HM3DGq injections failed and the animals did not enter synthetic torpor following CNO injection. These were excluded from the study (the success rate for inducing synthetic torpor was 80%). Some animals were excluded from the ischaemia-reperfusion study, due to time from heart excision to cannulation on the Langendorff apparatus being more than 2 minutes, more than 2 cannulation attempts or a heart rate of less than 200 bpm at the end of the equilibration period. 8 hearts were excluded from the heart infarction data set based on these criteria. We have updated the methods section of our manuscript to emphasise this.

(14) With small group sizes, it is better to show the data set variation with standard deviations rather than SEM.

We thank the reviewer for noting our mistake and have corrected it and/or reported individual data points.

(15) The precise P value for the infarct size reduction with torpor should be stated rather than p<0.05.

We have updated the manuscript and the figure legends to include the precise P values.

(16) Is there translational relevance of this work using an in vivo model of bona fide heart ischaemia-reperfusion injury?

We absolutely plan to complete those experiments, but feel they are beyond the scope of this initial reporting of the principle that synthetic torpor in the rat is cardioprotective.
